# A novel method for quantifying galleries of bark beetles and associates

**DOI:** 10.1093/jisesa/ieaf086

**Published:** 2025-10-07

**Authors:** J Forest Palmer, Jess A Hartshorn

**Affiliations:** Forestry and Environmental Conservation, Clemson University, Clemson, SC, USA; Forestry and Environmental Conservation, Clemson University, Clemson, SC, USA

**Keywords:** forest entomology, gallery mapping, digital imagery, pine engraver, *Pinus*

## Abstract

Bark beetles (Coleoptera: Curculionidae: Scolytinae) are some of the most destructive forest insect pests in the world. Methods literature is largely limited to trapping, including pheromones and volatiles, and remote sensing. Conversely, little work has been done on methods for quantifying bark beetle development under the bark. Assessing larval development involves marking and measuring each gallery using rulers or mapping tools, which takes much time and effort. We developed a novel graphite rubbing method on rice paper to quickly and accurately map larval bark beetle development under the bark of infested pine logs. We were then able to transpose the log surface onto a 2D plane, allowing photography and image analysis. We also describe alternative methods tested that were not successful. This method allows researchers to quickly collect data on gallery area and, therefore, larval development to have an additional piece of information to better predict and manage future outbreaks.

## Introduction

Bark beetles (Curculionidae: Scolytinae) are some of the most destructive forest insect pests in the world, with average annual impact among the highest of all forest disturbances (Grégoire et al. 2015). Bark beetles are both economically and ecologically important, affecting productive timber land like the Southeastern U.S. “timber basket” and specialized vulnerable habitats, such as those containing the western species North American whitebark pine (*Pinus albicaulis* Engelm.) (Raffa et al. 2015). Over the last century, land use practices, as well as changes to forestry markets and forest management, have resulted in significant shifts in bark beetle populations (Fettig et al. 2022). For example, increased temperatures and increased frequency and severity of storms due to climate change have been shown to increase bark beetle development rates and number of generations per year (Jönsson et al. 2009), change overwintering mortality and synchronicity of emergence (Creeden et al. 2014), and allow for movement of species into novel ranges and hosts (Dodds et al. 2018). As such, questions remain surrounding bark beetle monitoring and management strategies (Morris et al. 2017).

One key issue in the monitoring and management of bark beetles lies in their cryptic life cycle (Demidko et al. 2021). Trapping methods have been widely studied, and there are species-specific pheromones used to collect bark beetles for monitoring and outbreak predictions (Dodds et al. 2024). However, field validation of mathematical models created through trapping efforts are often biased (e.g., Goodsman et al. 2024) and trap catches do not necessarily align with tree damage or mortality (Barton 2015). Egg and larval galleries [ie tunnels created throughout the phloem of infested trees during reproduction, feeding, and overwintering (Raffa et al. 2015)] and specifically methods to quantify them, are much less studied. Because of their importance in bark beetle development, gallery abundance and length are frequently used in evaluating and predicting infestations and outbreaks (Anderbrant 1990, Rhodes et al. 1998, Davis and Hofstetter 2009). Gallery area has also been examined as a metric for identifying environmental effects from things like wildfire on bark beetle development and damage (Powell et al. 2012). Despite the complex yet direct relationship between gallery construction, bark beetle development, and survival, we were able to locate only 2 papers describing methods for measuring bark beetle galleries (Li and Li 2019, Smith et al. 2021), which used similar methods, including digital image processing, to reconstruct larval galleries on fallen pieces of bark. However, most papers which include gallery measurements in relation to bark beetle biology, development, or mortality do not report detailed methods for gallery measurements. The single paper specifically reporting methods of examining galleries (Li and Li 2019) does so on small pieces of fallen bark with clearly defined galleries and did not attempt to differentiate galleries from other naturally occurring marks on the bark, nor did they attempt to characterize galleries on logs. Furthermore, the photos of bark pieces in Li and Li (2019) do not account for the curvature of the bark, which inevitably distorts images and subsequent analysis.

Given the importance of bark beetle gallery quantification in evaluating population growth and dynamics, and the current dearth of literature focusing on this issue, we set out to create and describe a quick yet effective method for quantifying bark beetle galleries in real-world scenarios relevant to managers and researchers. Our objective is to describe a novel method for quantifying bark beetle galleries that can be used by researchers to develop new predictive models, monitor bark beetle populations, and predict future infestations or outbreaks.

## Log Preparation

We selected 12 loblolly pine (*Pinus taeda* L.) trees across four sites (*n *= 3 per site) in the Clemson Experimental Forest (Pickens County, South Carolina, United States ) for destructive sampling (Table 1). Most stands were overly dense, between 14.9 and 18.6 m^2^ (160 to 200 ft^2^) basal area (BA), consisting mostly of planted even-age loblolly pines (Table 1) with a moderate woody understory consisting of sweetgum (*Liquidambar styraciflua* L.), American beautyberry (*Callicarpa americana* L.), and various forbs such as dogfennel [*Eupatorium capillifolium* Lam. (Small)]. Stands received a prescribed burn in February 2022 as part of the Clemson Experimental Forest management plan. Burns were conducted by certified prescribed fire managers and maintained expected characteristics (eg flame length) for prescribed fire in southern pines (Lotti 1962). Burned trees were selected to ensure infestation by bark beetles and their associates. Immediately following the burns, we felled three trees per site and left them exposed on the ground at the site for 30 d. At the end of 30 d, we cut each tree into quarters relative to their individual height and brought the middle 0.5-m section from each quarter to the Clemson Cherry Farm at Clemson University to rear out bark beetles (*n* = 48 logs). The low and two middle sections were placed into 60.96 × 60.96 × 91.44 cm RESTCLOUD butterfly habitat cages, with the top sections being placed in 39.87 × 39.87 × 59.95 cm cages (as the top sections were significantly smaller). Logs were left in the rearing (habitat) cages for 90 d to allow any developing insects to emerge, after which time logs were dissected as described below. A random subsample of 10 logs was selected for phloem thickness (mm) measurements using Control Company (Houston, Texas, United States) digital calipers to measure the phloem thickness at the top and bottom of each log.

**Table 1.** Site coordinates in the Clemson Experimental Forest (Pickens County, South Carolina, United States) along with burn dates and basal area (m^2^)

Site	Latitude, longitude	Basal area (m^2^)	Year planted
**Six mile**	34.744246, −82.837555	14.9	2000
**Queen street**	34.638585, −82.823563	18.6	1986
**Vet school**	34.641350, −82.813250	8.4	1984
**Issaqueena**	34.741910, −82.865851	16.7	1986

## Procedure

### Gallery Mapping

**Fig. 1.**
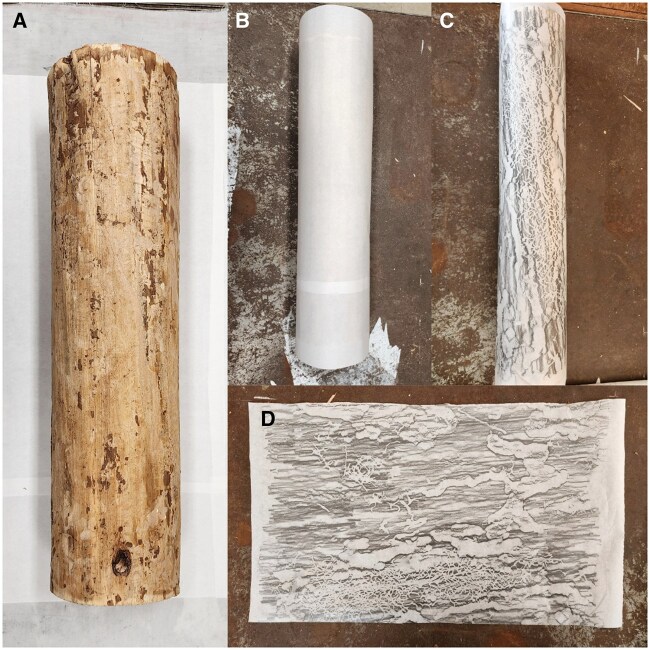
A 0.5 m loblolly pine log debarked A), wrapped in rice paper B), and rubbed with graphite pencil C). The rice paper with the graphite rubbing is then unwrapped and laid flat to be transposed for image analysis.

To ensure that galleries were left intact, we carefully removed the bark by hand using 1- and 1.5-inch Ace chisels and a brush to remove debris surrounding the galleries (Fig. 1A). Once galleries were exposed, we wrapped a large (30.48 × 45.72 cm) piece of Master’s Touch rice paper around the log so that the paper was snug against the surface and bark beetle galleries could be seen through the paper (Fig. 1B). We then cut the rice paper to the appropriate length depending on the circumference of the log and taped it together at the ends to secure it.

**Fig. 2.**
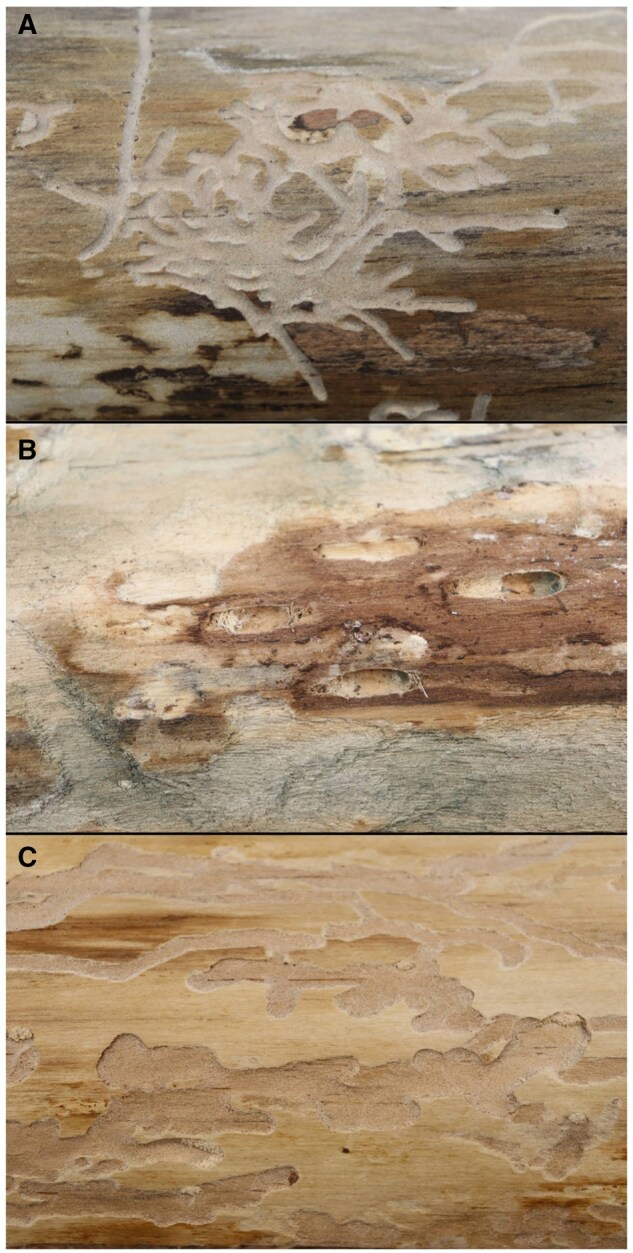
Photo plate showing *Ips* A), pine weevils B), and *Monochamus* C) galleries on the surface of debarked pine logs.

Once the rice paper was secured around the log, we created a rubbing over the entire surface of the paper using a 6B or 8B Master’s Touch graphite refill (Fig. 1C). We then unwrapped the rice paper from the log and inspected it carefully to ensure that the white lines created with the rubbing were in fact bark beetle galleries (Fig. 1D). After comparing the rice paper to the log, we laid the paper flat underneath a 1-cm gridded transparency sheet. We then traced the galleries onto the transparency sheet using one of four colors of dry erase markers and using fine-tip Sharpie markers for borders, depending on the genus: green indicated *Ips* (Fig. 2A), blue indicated pine weevils (*Pachylobius* LeConte*, Pissodes* Germar*, Hylobius* Germar, and *Naupactus* Dejean) (Fig. 2B), and red indicated *Monochamus* longhorn beetles (Coleoptera: Cerambycidae) (Fig. 2C). Galleries were identified using visual cues such as size, shape, and contents; *Ips* bark beetles create small (∼1 mm wide) galleries in a Y or H shape (Coyle et al. 2016) which are filled with sawdust-like frass, while *Monochamus* larvae are much larger (3+ mm wide) and create winding galleries in a D or S shape which are filled with excelsior (Pershing and Linit 1986, Togashi et al. 2005). Non-scolytine weevils (e.g., *Hylobius pales*) feed on the inner bark and cambium of pines and thus do not leave noticeable galleries in the sapwood until pupation. Instead, pupal cases were identified by their oval shape also surrounded by excelsior (Maine Department of Agriculture 2000).

### Image Analysis

**Fig. 3.**
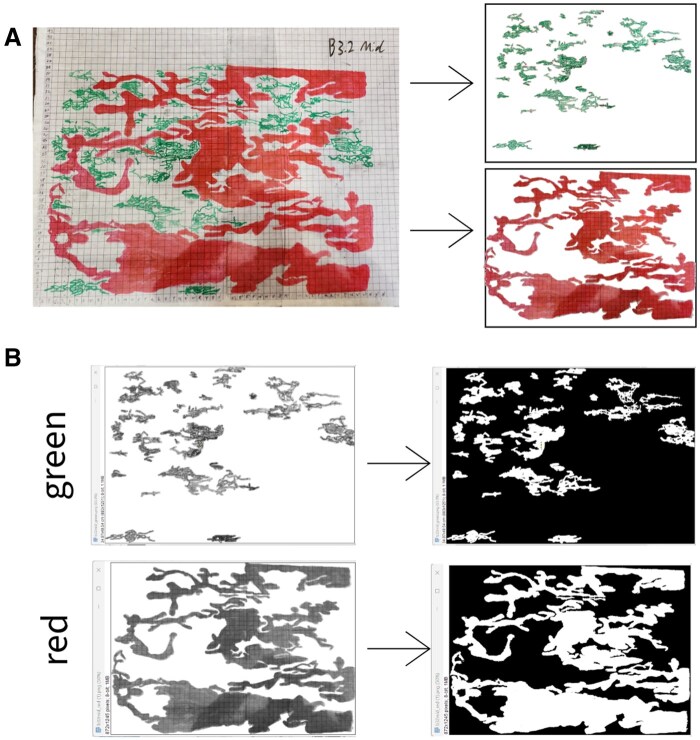
Transposed galleries of *Ips* (green) and *Monochamus* (red) photographed and split into separate colors (green—*Ips* bark beetles and red—*Monochamus* longhorn beetles) A) which were then converted to an 8-bit image and the ‘intermodes’ threshold applied B).

After galleries were completely transposed onto transparency sheets, we took photos of each sheet against a plain white background and cropped each image to include only the surface area of the log. Using Adobe Capture v9.1.1 (Adobe, Inc., San Jose, California, United States), we selected one color at a time and created individual images for each genus described above (Fig. 3A). We then opened each image in ImageJ v1.54k (Schneider et al. 2012) and set the scale for analysis by drawing a line segment over one side of a gridded cell in the image and setting that line segment to be equal to a 1 cm gridded cell. After the scale was set, we converted images to 8-bit, which creates a grayscale image showing galleries as dark-colored on a white background (Fig. 3B). We then used the ‘auto threshold’ option in ImageJ which identifies the threshold of gray at which point a pixel will be made white or black. Specifically, we used the “Intermodes” method, which calculates the mean of all pixels with gray values above and below the auto-threshold, further adjusting the threshold to be halfway between these two means. This produced the black and white image most congruent with the original gallery image. From the 8-bit converted image with thresholds set, we then selected “Analyze” and “Measure” which produces an output of the total area of galleries.

## Discussion

This paper demonstrates a novel and highly accurate method to transpose bark beetle galleries on infested logs for quantification and digital analysis of egg and larval galleries. Prior to this, little information existed that gave researchers and land managers a quick and efficient way to measure these galleries (Li and Li 2019). Prior to the graphite pencil rubbing on rice paper, we attempted several other methods of transposing galleries. The first method we attempted involved pouring liquid latex (FX Latex, Morton, Pennsylvania, United States) over the excavated galleries to create a mold of the negative space. While in theory this method would work, the liquid latex did not sufficiently stick to the log and only created a thin layer. To do this method successfully, one would need an additional mold surrounding the entire log so that liquid latex could fill the space between the log and the mold. This would be too expensive and time-consuming to do for every log because, even if logs were the same size and a single mold could fit around all logs, latex molds have a shelf life and would need to be remade after 5 to 10 logs.

We also attempted wrapping logs in multiple materials, including Saran wrap, plastic transparency sheets, and thin cotton fabric (ie cut pillowcases). The Saran Wrap and pillowcases were not sturdy enough to remain taut during the rubbing process, and they had to be continuously repositioned while also not producing good results. While others were able to use plastic transparency sheets to map entry holes (Takei et al. 2021) and galleries (Smith et al. 2021), the plastic transparency sheets we used were not thin and flexible enough to adequately mark galleries using a rubbing utensil. We also attempted to simply draw the galleries on the transparency sheets wrapped around logs. However, the sheets would not lay flat, and therefore we could not accurately draw galleries in relation to other bumps and marks on the log that were raising the transparency sheets.

Rice paper is available in large sheets at any hobby or craft store, is inexpensive, and is flexible and thin enough to sit flat across the entire surface of the log, even with bumps, branch collars, and other anomalies creating an uneven surface. We also attempted other utensils for rubbing galleries, including crayons, #2 pencils, colored pencils, and markers. Each resulted in a variety of issues; for example, crayons simply marked the entire log and did not provide enough distinction between galleries and other marks or bumps along the surface. Similarly, #2 pencils (which contain a mixture of graphite and clay), colored pencils, and markers resulted in a single sheet of color with little differentiation between galleries and the log surface. Lightly rubbing with a graphite refill produces an image that has clear differentiation between anomalies of the log surface and galleries created by bark beetles. Graphite refills are also inexpensive and readily available at any hobby or craft supply store. Charcoal was also tested but proved too soft to make consistent rubbings and tended to break apart during the rubbing process.

Gallery area is just one metric used to assess bark beetle populations (e.g., Hayes et al. 2008). Area measurements as we’ve presented are the simplest measurements available using the base ImageJ package. Additional plugins like AnalyzeSkeleton (Arganda‐Carreras et al. 2010) can allow researchers to convert galleries into lines and then output the length of each segment as well as combined segments. This plugin specifically would allow observers to compartmentalize galleries based on the location of eggs or nuptial chambers as well. Additionally, the ImageJ operation ‘Analyze Particles’ allows users to identify individual points in an image and could potentially be used to quantify egg niches or nuptial chambers in the appropriate scenarios.

One factor that may pose an issue is phloem thickness, which has been shown to directly affect the size and development of bark beetles in pine (Haack et al. 1987). All of our pine logs had phloem 1.1 to 1.6 mm thick, which allowed for bark beetles to create clear engravings on the xylem of the log. In trees with thicker phloem, bark beetles may feed throughout the phloem without making obvious marks on either the inner bark or the log surface. If phloem thickness is measured ahead of time, log dissections can be modified so that more phloem is left intact, and engraving on the xylem is not necessary to create a rubbing. In heavy infestations, though, the bark may completely slough off, and the phloem may be nearly nonexistent. In these cases, it would also be difficult, if not impossible, to measure bark beetle galleries by traditional methods as well.

Egg and larval galleries are important metrics for assessing bark beetle population dynamics, development, fitness, and survival (Anderbrant 1990, Rhodes et al. 1998, Davis and Hofstetter 2009). The method we described here can be used by researchers to quantify egg and larval galleries of bark beetles, which can then be used to update or modify existing predictive models of development or outbreaks (e.g., Esch et al. 2016, Podlaski et al. 2020). This method is also useful in creating visual representations of bark beetle galleries, which are normally only viewed piecemeal on the curved surface of a log. We also demonstrate the novel benefits of combining art and science and hope that other researchers see this as an opportunity for new collaborations with innovators outside of their specific scientific field.
